# Unmasking tuberculosis: Isolated hilar adenopathy in an asymptomatic immunocompetent adult

**DOI:** 10.5339/qmj.2024.qitc.18

**Published:** 2024-03-25

**Authors:** Aasir M. Suliman, Mushtaq Ahmad, Mustafa A. Al-Tikrity, Ibrahim Rashid

**Affiliations:** 1 Pulmonology Department, Hamad General Hospital, Hamad Medical Corporation, Doha, Qatar Email: asuliman4@hamad.qa

**Keywords:** Tuberculosis, TB, Tuberculous hilar adenopathy, hilar adenopathy

## Background

Isolated mediastinal lymphadenopathy is a common manifestation of tuberculosis (TB) in children and adults with human immunodeficiency virus (HIV) infection. However, it is rare in immunocompetent adults without a parenchymal lung lesion, and presents a diagnostic problem due to the low diagnostic yield of sputum examination and the wide range of differential diagnoses.^1,2^

## Case Presentation

**Figure 1.**
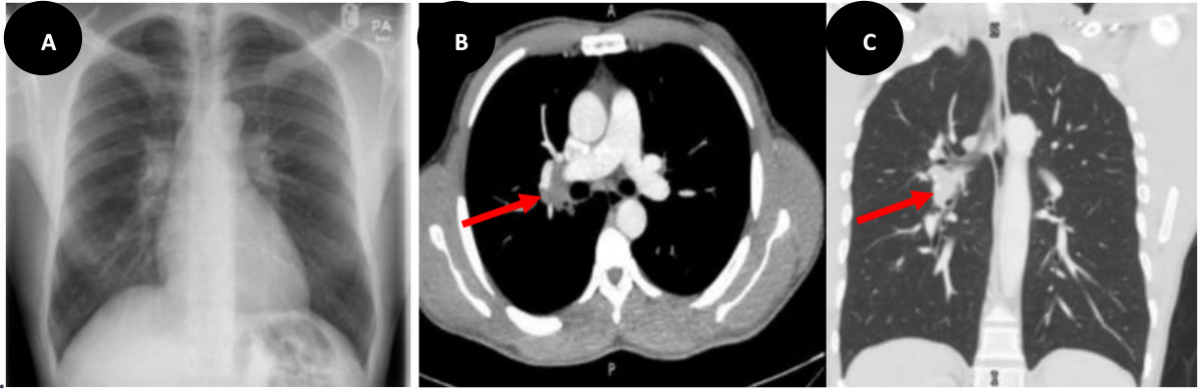
A chest radiograph demonstrates a prominent hilar regions (A). An axial and coronal CT thorax views showing single enlarged right hilar lymph node with otherwise clear both lungs fields (B & C).

A 36-year-old, previously healthy and asymptomatic male was found to have prominent hilar shadows during a routine chest X-ray (Figure 1A) conducted for immigration screening. Subsequent computed tomography (CT) chest imaging confirmed a single enlarged right hilar lymph node (Figure 1B and C) without additional parenchymal abnormalities. Sputum samples for acid-fast bacilli (AFB) smear, AFB polymerase chain reaction (PCR), and TB culture were all negative. Hilar tuberculous adenopathy was conclusively confirmed by endoscopic bronchial ultrasound (EBUS) with a positive fine needle aspiration (FNA) for AFB PCR. Notably, the serum HIV antigen/antibody test showed a non-reactive result. Consequently, the patient began a 6-month course of anti-tubercular treatment.

## Conclusion

Isolated tuberculous mediastinal lymphadenopathy in immunocompetent adults is uncommon and may be underdiagnosed, given that patients are often asymptomatic until there is mass effect and compression of adjacent structures.^2^ The differential diagnosis of a mediastinal mass is broad and poses a common challenge in clinical practice. This case highlights the importance of considering TB as a potential etiology in adults with mediastinal lymphadenopathy without lung parenchymal involvement, even if the patient is asymptomatic or immunocompetent. Early recognition is crucial to initiate prompt treatment, avoid unnecessary invasive procedures, and achieve better outcomes.

## Conflict of Interest

The authors declare that they have no known competing financial or personal interests.
